# Objectively measured physical activity following lumbar decompression surgery: systematic review and meta-analysis

**DOI:** 10.1038/s41598-026-44749-1

**Published:** 2026-04-01

**Authors:** Sree Kanakala, Alisha Mahmud, Iihan Ali, Hassan Tahir, Riese Patel, Milos Brkljac, Tim Lindsay

**Affiliations:** 1 School of Medicine, Imperial College London, London, UK; 2 Faculty of Medicine, University of Birmingham, Birmingham, UK; 3 MSk Lab, Imperial College London, London, UK

**Keywords:** Lumbar decompression, Physical activity, Accelerometer, Step count, Spine surgery, Diseases, Medical research, Neurology

## Abstract

Lumbar decompression surgery reliably improves pain and disability in lumbar disc herniation and spinal stenosis, but whether these symptomatic gains translate into greater free-living physical activity (PA) remains unclear. This systematic review and meta-analysis evaluates postoperative changes in objectively measured PA, and its association with patient-reported outcome measures (PROMs). PubMed, MEDLINE, EMBASE and Scopus databases were searched to identify studies reporting objectively measured PA after lumbar decompression, including laminectomy, laminotomy, foraminotomy and discectomy. Ten studies (*n* = 549) met inclusion criteria, with six (*n* = 199) included in a random-effects meta-analysis of standardised mean change (SMC) in PA volume at 3 and 6 months. Meta-analysis demonstrated small, non-significant improvements in PA volume at 3 months (SMC 0.26; 95% CI -0.16, 0.69) and 6 months (SMC 0.25; 95% CI -0.13, 0.63). PA volume followed a consistent pattern: an early postoperative decline, followed by recovery to pre-operative levels by 3–4 months. There were limited behavioural shifts towards higher activity intensities. Correlations between PA and PROMs were weak and inconsistent. Objectively measured PA shows limited and heterogeneous recovery after lumbar decompression despite marked symptomatic improvement. Further work should standardise PA measurement and evaluate behavioural interventions to support postoperative activity.

**Supplementary Information:**

The online version contains supplementary material available at 10.1038/s41598-026-44749-1.

## Introduction

Lumbar disc herniation (LDH) and lumbar spinal stenosis (LSS) are leading causes of radiculopathy worldwide and are associated with a significant symptom burden^1,2^. LDH and LSS can cause a wide range of symptoms, including pain, sensory disturbances, and motor deficits, all of which can diminish functional capacity and thus quality of life^3,4^. Lumbar decompression is an established treatment for patients with persistent or severe symptoms^5,6^. The literature on postoperative patient-reported outcome measures (PROMs) is extensive and shows marked improvements in pain and disability^6–8^. However, it remains unclear whether the improvements in symptoms following surgery translate into greater free-living physical activity (PA) levels.

Beyond surgery-induced symptomatic relief, physical activity must be recognised as a life-conserving behaviour in and of itself, and thus an important outcome measure. In a large population-based study of over 96,000 participants, Strain et al. demonstrated that both PA volume and intensity were independently associated with all-cause mortality^9^. Similar associations have been observed with other disease states, including obesity, depression and anxiety^10,11^, underpinning the importance of the restoration and preservation of PA to the holistic health of the patient. Despite the sweeping benefits of PA, relatively little is known about the effects of decompression on physical activity levels. Even less is known about the intensity distribution of physical activity following surgery. This represents a core knowledge gap in our evaluation of recovery following lumbar decompression.

PROMs have long formed the cornerstone of postoperative assessment in spine surgery, though they possess an inherent ceiling effect and limited granularity^12^. For example, they cannot distinguish between high-functioning patients and have been shown to underestimate the burden of motor deficits in LDH^3,12^. Consequently, PROMs do not fully capture functional recovery following surgery. Recent advances in wearable technology, including accelerometers and smartphones, enable the objective measurement of physical activity in free-living environments^13^. Wearables address these specific limitations of PROMs and yield continuous, high-resolution data^14^. Crucially, they allow activity to be delineated into two distinct elements, volume and intensity, providing a more nuanced evaluation of postoperative function than PROMs alone^15^.

To date, no systematic review or meta-analysis has synthesized the evidence on device-measured PA following lumbar decompression. Physical activity outcomes represent an important gap, given the increasing adoption of wearables, and the substantial clinical burden of LDH and LSS. The present systematic review and meta-analysis addresses this by evaluating changes in free-living PA volume and intensity following lumbar decompression, and by examining correlations between subjective and objective measures of PA. We hypothesised that decompression would lead to measurable improvements in free-living physical activity volume and intensity. Additionally, we hypothesised that objectively measured physical activity would correlate with postoperative PROM scores, and where reported, with changes in PROMs from baseline.

## Methods

### Protocol and registration

This study was prospectively registered with PROSPERO (CRD420251124422) and was conducted in accordance with PRISMA guidelines^16^.

### Search strategy, study selection & data extraction

The literature search was conducted on 25th August 2025 across PubMed, MEDLINE, EMBASE, and Scopus databases from 01/01/2000–15/08/2025. The detailed search strategy is available in Supp. Table 1, with the PRISMA flowchart outlining the study selection process in Supp. Fig. 1. Original, peer-reviewed studies published in English that examined PA in adults undergoing lumbar decompression were included. Lumbar decompression procedures included any procedure aimed at relieving neural compression, such as laminectomy, laminotomy, foraminotomy and discectomy with or without fusion. Only studies that reported raw PA values at defined post-surgical timepoints or within clearly specified postoperative intervals were included in the meta-analysis. All studies were included in the qualitative synthesis. Studies involving children (< 18 years), reviews, editorials, and case reports were excluded, as detailed in Supplementary Table S2. Initial screening and full-text assessment were performed using Covidence software for duplicate removal, and the results were independently reviewed by three reviewers (AM, IA, HT). Any disagreements were resolved by consensus with SK. Relevant data from each included study were manually extracted on Excel spreadsheets, capturing information on study characteristics (title, authors, country of publication, publication date), surgical technique, demographic details and study conclusions. This was done by three independent researchers (AM, IA, HT). For studies with missing data, corresponding authors were contacted. Further data were obtained for two studies^17,18^.

### Critical appraisal

Three independent reviewers (AM, IA, HT) assessed the risk of bias for each study using the Risk of Bias in Non-Randomised Studies of Interventions (ROBINS-I) for non-randomized studies^19^. The quality of evidence was assessed using the Oxford Centre for Evidence-Based Medicine (OCEBM) Levels of Evidence^20^.

### Statistical analysis

Data preparation was undertaken using Excel, followed by statistical analysis and forest plot synthesis in R (Version 4.4.3) utilizing the *meta* package^21^. A random-effects meta-analysis was conducted to calculate the pooled standardized mean change (SMC) in physical activity volume at different postoperative timepoints. SMC > 0 showed an increase in physical activity. The results of the meta-analysis were visualized using forest plots that illustrated effect sizes, 95% confidence intervals (CIs), and pooled estimates. Heterogeneity across studies was quantified using the I² statistic. Statistical significance was defined at a threshold of *p* < 0.05.

## Results

### Study characteristics

Of 1,566 records identified, 224 duplicates were removed. 1,342 records were screened by title and abstract, of which 1,320 were excluded. The remaining 22 full-text articles were assessed for eligibility, and 12 were excluded with reasons documented in Supp. Fig. 1. Ten studies were included in this review^17,18,22–29^. Study characteristics, including design, country, sample size, and follow-up duration, are summarized in Table 1. A total of 549 patients were included. The pooled mean follow-up was 9.2 (SD 3.77) months. The pooled mean age was 62.8 (SD 12.68) years and pooled BMI was 30.1 (SD 6.56) kg/m^2^. Male patients accounted for 49% of the cohort. The included studies comprised eight prospective cohort studies, one retrospective cohort study and one secondary analysis of a RCT. Publication frequency increased over the last decade (Supp. Fig. 2). Most studies originated in North America and Europe, with the United States contributing the most (*n* = 5). The risk of bias assessments across all studies are summarized in Supp. Figs. 3 & 4. Details of physical activity monitoring, such as monitor type, wear duration and wear location, are detailed in Table 2.

**Table 1:** Characteristics of included studies evaluating objectively measured physical activity following lumbar decompression. Sample sizes, study design, level of evidence, patient demographics, procedure type, and follow-up durations are presented.

Author	n	Study design	Level of evidence	Country	Age	BMI	% Male	Type of lumbar surgery	Follow-up period
Aubry 2021	29	Prospective, observational study	2b	Switzerland	56.6 (11.7)	26.5 (5.5)	37.9	Discectomy, Decompression, Laminotomy, Foraminotomy	6 and 12 weeks
Bienstock 2022	25	Prospective cohort study	2b	USA	64.5 (8.8)	29.2 (4.7)	55.0	Laminectomy ± Fusion	6 months
Chauhan 2024	52	Retrospective cohort study	2b	USA	64.4	NR	55.8	Decompression, Fusion	40 weeks
Coronado 2021	248	Secondary analysis of an RCT	2b	USA	62.2 (11.9)	32.4 (6.6)	49.0	Laminectomy ± Fusion	12 months
Inoue 2020	60	Prospective observational study	2b	Japan	70.7 (8.4)	24.5 (4.4)	50.0	Decompression, Fusion	1, 3, 6, and 12 months
Mobbs 2016	30	Prospective observational study.	2b	Australia	42.6 (10.3)	NR	60.7	Laminectomy, Discectomy, Fusion	1, 2 and 3 months
Scheer 2017	32	Prospective pilot study	2b	USA	58.1 (12.7)	NR	50.0	Microdecompression, Foraminotomy, Fusion, Instrumentation	6 weeks, 3 months, and 6 months
Schulte 2010	50	Prospective study	2b	Germany	69.3 (7.5)	28.7 (4.4)	48.9	Decompression	3 and 12 months
Smuck 2018	38	Prospective cohort study.	2b	USA + Canada	70.1 (8.9)	28.4 (6.2)	39.3	Decompression	6 months
Stienen 2022	18	Prospective observational feasibility study	2b	USA	57.1 (14.9)	28.8 (4.9)	46.7	Decompression, Discectomy	1, 2, 4, 8, 12 weeks, 6 months, 12 months

**Table 2:** Device characteristics, wear protocols, and key physical activity findings across included studies. Wear location, monitoring duration, and extracted metrics of physical activity volume and intensity are summarised alongside each study’s principle conclusions.

Author	Device	Wear location	Wear criteria	Measures of PA volume	Measures of PA intensity	Main conclusions
Aubry 2021	ActiGraph GT3X+	Right side of waist	3–7 consecutive days at each timepoint	Steps/day	ST, LPA, MPA, VPA, MVPA (mins/week)	Steps/day and MVPA increased within 12 weeks but remained below healthy controls. Improvements in ODI and HRQoL correlated with PA at 6 weeks only.
Bienstock 2022	Fitbit Flex 2	Non-dominant wrist	Continuous	Daily step counts, Daily aggregate median steps and individual visit-specific median steps	NR	Recovery after laminectomy progressed through three phases - rapid early gains, gradual recovery, and plateau by four months. Step counts correlated with PROMs at limited timepoints. Only PROMs showed significant long-term improvement.
Chauhan 2024	iPhone Health App	Smartphone carrying	Continuous	Steps per day, Rate of change in steps per day	NR	Fusion patients showed greater preoperative decline, slower postoperative recovery, and larger secondary decreases in activity compared to decompression patients.
Coronado 2021	ActiGraph GT3X+	Right Hip	7 consecutive days at each timepoint	Mean activity counts/minute	NR	Higher postoperative resilience and pain self-efficacy predicted superior 12-month PROMs, but not physical activity.
Inoue 2020	Actigraph^®^ Micro-Motion	Non-dominant Wrist	7 consecutive days at each timepoint	Mean activity counts/minute	NR	Activity declined in the first postoperative month but recovered by three months. PROMs improved earlier.
Mobbs 2016	Fitbit Zip	Belt, Waistband, Pant pockets	7 consecutive days at each timepoint	Steps per day, distance travelled per day, calories burned per day	NR	Steps/day and distance increased significantly by three months, alongside improvements in VAS, ODI, and SF-12. No significant correlation was found between changes in PROMs and activity measures.
Scheer 2017	Fitbit Flex	Wrist	2–4 weeks preoperatively, 5 months postoperatively	Steps per day, maximum hourly steps	ST, LPA, MPA, VPA (mins/day)	Higher preoperative step counts correlated with better ODI and PCS scores. Only two of 11 patients showed significant postoperative gains, while six declined in activity.
Schulte 2010	StepWatch Activity Monitor	Ankle	7 consecutive days at each timepoint	Number of gait cycles	Number of gait cycles per minute.	Most patients improved within the first three months, with outcomes stabilising thereafter and similar across sexes.
Smuck 2018	ActiGraph GT3X+	Right Hip	7 consecutive days at each timepoint	Total daily activity counts	ST, LPA, MPA, VPA (mins/day)	At six months, patients improved in pain, disability, and functional capacity, but not in free-living activity levels.
Stienen 2022	Xiaomi Mi Band	Wrist	Continuous wear from pre- to 12 months post-op	Steps per day	NR	Steps dropped sharply post-op, returning to baseline by 8–12 weeks. PROMs improved but correlated weakly with activity.

### Physical activity volume

**Fig. 1:**
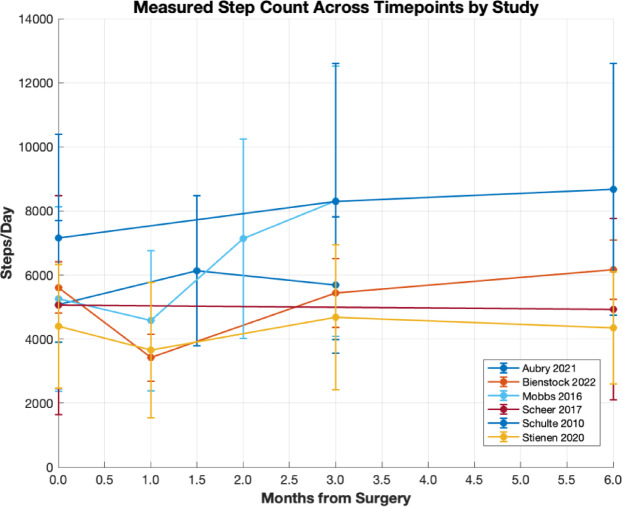
Mean daily step counts across postoperative timepoints by study following lumbar decompression surgery. Error bars represent standard deviations where reported.

Eight studies assessed physical activity volume, of which six measured daily step count. Figure 1 summarises the mean step count trajectories of all relevant studies in the first six postoperative months. Across studies, physical activity volume showed a consistent postoperative trajectory: an early decline immediately after surgery, followed by a rapid rise in daily activity during the next few weeks and plateauing around preoperative levels by three to four months^17,18,26^. Some studies observed modest sustained gains or secondary declines beyond this period^22,27^. Measures other than step count, such as accelerometer-derived active counts and gait cycles, demonstrated comparable trends^25,28^. Overall, free-living physical activity volume improved following lumbar decompression, though recovery patterns varied between individuals and measurement methods.

### Physical activity intensity

Three studies assessed activity intensity, of which two examined moderate-to-vigorous physical activity (MVPA) and two light physical activity (LPA) and sedentary time (ST). MVPA generally increased early after surgery but remained below reference levels^22,29^. Light physical activity and sedentary time stayed stable, and showed minimal overall shifts toward higher-intensity behaviour^27,29^. MVPA, LPA and ST trajectories varied between individuals. Intensity-based outcomes were infrequently reported, limiting cross-study comparison.

### Correlation between subjective and objective measures of physical activity

Five studies explored associations between objectively measured physical activity and PROMs. Three studies correlated raw postoperative objective PA values with PROM scores^17,18,24^. Studies and PROMs significantly correlating with PA volume, including the direction of association, are summarised in Table 3. Across studies, higher step counts generally correlated with lower disability and better physical function scores in the early postoperative period^17,18^. Moderate correlations were observed between step count and ODI or SF-12/SF-36 PCS scores within the first three months, though these relationships weakened or disappeared by one year. Associations with pain scores were inconsistent^22^. No independent relationship was found between objective PA and psychosocial factors, such as pain self-efficacy and resilience, at 12 months post-surgery^24^. Two studies correlated changes in PROMs with changes in PA over time^22,26^. There was not a robust, consistent relationship between subjective function and objective physical activity.

**Table 3:** Summary of patient-reported outcome measures showing significant correlations with objectively measured physical activity volume at different postoperative timepoints.

Timepoint	PROMs showing significant correlation with PA volume	Direction of correlation	Source
Preoperative	Leg-pain VAS, ODI	Inverse	Schulte et al.^28^
2 weeks	ODI	Inverse	Bienstock et al.^18^
1 month/6 weeks	ODI, SF-12 PCS, SF-36	ODI inverse, SF-12/36 positive	Bienstock et al.^18^, Aubry et al.^22^
3 months	ODI, SF-12 PCS	ODI inverse, SF-12 positive	Bienstock et al.^18^, Stienen et al.^17^, Schulte et al.^28^
6 months	SF-12 PCS	Positive	Bienstock et al.^18^
12 months	Leg-pain VAS, ODI	Inverse	Schulte et al.^28^

### Measurement device types

Device type, wear location, and wear-time criteria are summarised in Table 2. Studies using research-grade accelerometers (*n* = 5) tended to specify explicit wear-time criteria to ensure data validity. Studies using consumer-grade devices (*n* = 4) or smartphones (*n* = 1) relied on habitual or passive wear without defined thresholds. Wear locations varied across studies: waist/hip (*n* = 3), wrist (*n* = 4), ankle (*n* = 1), and passive carrying (*n* = 2). Variability in device type, placement, and adherence protocols likely contributed to heterogeneity in reported activity outcomes.

### Meta analysis

Six studies (*n* = 199) were included in the quantitative synthesis. Outcomes were expressed as standardised mean change (SMC) in PA volume. Data were reported as either daily step count or mean activity count (MAC), both considered surrogate measures of activity volume.

**Fig. 2:**
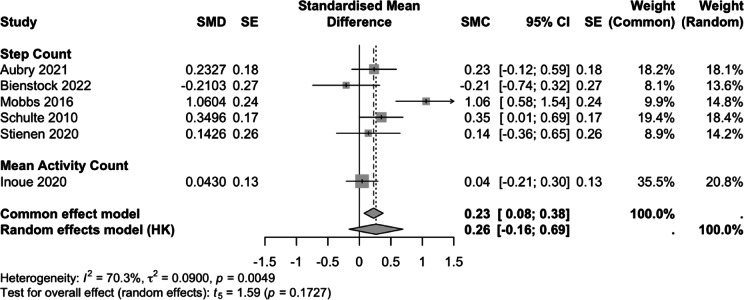
Forest plot of standardized mean change (SMC) in objectively measured physical activity volume at 3 months following lumbar decompression surgery. Individual study estimates, pooled effects and heterogeneity statistics are displayed.

**Fig. 3:**
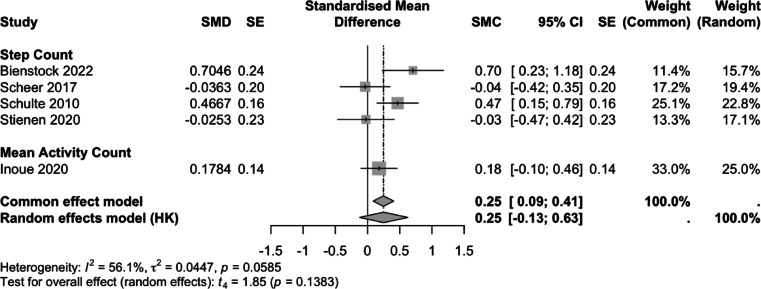
Forest plot of standardized mean change (SMC) in objectively measured physical activity volume at 6 months following lumbar decompression surgery. Individual study estimates, pooled effects and heterogeneity statistics are displayed.

At 3 months postoperatively, five studies reported step count and one reported MAC (Fig. 2). The pooled SMC was 0.26 (95% CI -0.16, 0.69; *p* = 0.17). Heterogeneity was moderate (I² = 70.3%, *p* = 0.005). Bienstock was an outlier, as the only study to show a negative SMC at 3 months. At 6 months postoperatively, four studies reported step count, and one study reported MAC (Fig. 3). The pooled standardized mean change was 0.25 (95% CI: -0.13, 0.63; *p* = 0.14), with moderate heterogeneity (I² = 56.1%, *p* = 0.059). At both timepoints, the limited number of studies precluded sensitivity analyses.

## Discussion

### Summary of findings

To our knowledge, this is the first systematic review and meta-analysis to quantitatively assess objectively measured physical activity profiles following lumbar decompression surgery. Across ten studies and over 500 patients, our findings show that improvements in symptoms and patient-reported outcomes are not accompanied by increases in free-living physical activity, with postoperative activity generally returning to preoperative levels or only modestly above.

### Physical activity volume

Across studies, PA volume followed a broadly consistent postoperative trajectory. Most cohorts exhibited an initial decline in activity during the immediate postoperative period, followed by a progressive increase over subsequent months. By three to six months, several studies reported activity levels exceeding preoperative baselines^26,28^. Early inactivity is expected and likely multifactorial, driven by postoperative pain, fatigue and lumbar surgery-specific activity restrictions that typically extend for 6 weeks^30^. However, evidence suggests that early mobilisation protocols can accelerate recovery and increase ambulation in the first six postoperative weeks^31^. In a randomised trial, Newsome et al. found that initiating exercise within two hours of lumbar microdiscectomy halved the time to independent mobility and shortened return-to-work by two weeks^32^. Although these findings plausibly reflect increased PA, the effect of early mobilisation protocols on free-living physical activity has not been directly measured, and therefore remains uncertain. Further work is needed to assess the effect of such interventions on free-living PA in the immediate postoperative period.

Another likely contributor to postoperative inactivity is the degree and duration of dysfunction prior to surgery. Patients who are severely incapacitated, or remain so for prolonged periods prior to surgery, may experience sarcopenia, defined as the progressive loss of skeletal muscle mass and strength^33^. Sarcopenia has been shown to be difficult to reverse, particularly in older adults, as muscle mass gains remain limited even with targeted resistance training^34^. In patients undergoing lumbar decompression, who are often elderly, and experience extended preoperative inactivity, these effects may be amplified. The timing from symptom onset to surgery therefore warrants closer examination as a determinant of postoperative PA, particularly given the prolonged waiting times for elective spinal procedures in global healthcare systems^35^.

A clinically relevant observation is that PA volume recovery patterns are highly variable between individuals. In a cohort of 22 patients, Scheer et al. found that 11 increased their step count, 5 showed no change and 6 recorded lower step count at 6 months compared to baseline^27^. Heterogeneity in recovery may reflect, in part, the lack of structured promotion of physical activity within spinal postoperative care. Freene et al. reported that only 54% of physiotherapists routinely promoted PA to their patients, highlighting a tendency to neglect physical activity as an outcome during the rehabilitation pathway^36^. Standardising postoperative counselling and integrating explicit PA targets into recovery protocols could help reduce variability, and encourage more consistent engagement in free-living activity following decompression.

Overall, the gradual, non-linear trend in PA volume aligns with trajectories observed in other orthopaedic populations, including hip and knee arthroplasty, where early reductions in activity are often succeeded by robust functional gains^37^. Within arthroplasty cohorts, long-term postoperative activity profiles, often extending beyond two years, are well documented^38^. In contrast, most studies of lumbar decompression assessed outcomes only up to six months. Extended follow-up is feasible in the lumbar decompression field, as shown by RCTs collecting PROMs and clinical outcomes for up to a decade^39^. The lack of physical activity data beyond a year therefore limits our ability to characterise longer-term PA profiles, and it remains unclear whether improvements regress, plateau or continue past twelve months.

### Physical activity intensity

Physical activity intensity is a critical yet underappreciated dimension of postoperative recovery. Cardiometabolic health, bone density, and psychological wellbeing all depend on achieving sufficient time in moderate-to-vigorous activity^40^. Additionally, Strain et al. demonstrated that PA intensity is independently associated with all-cause mortality, with higher-intensity activity conferring additive benefits even when total volume is controlled for^9^. At low overall activity levels, individuals who spent an additional 10% of their time in MVPA have a 30% lower mortality rate than their peers - a difference equivalent to replacing a 12-minute stroll with a brisk 7-minute walk. Given small increases in activity intensity yields life-preserving benefits, every minute of higher-effort movement matters in recovery^41^. Therefore, intensity of PA is just as worthy of study as volume of PA.

We hypothesised that postoperative reductions in pain and disability, as reflected by improvements in PROMs, would be accompanied by a behavioural shift in activity intensity. Specifically, we anticipated reductions in sedentary time and increases in MVPA. However, the available evidence did not support this expectation. Only two studies examined activity intensity, and their findings were variable: while some patients demonstrated reductions in sedentary behaviour or increased time spent in higher-intensity activity, others showed minimal or no change. Evidence on the determinants of postoperative PA intensity remains extremely limited in the decompression population. Factors influencing PA volume, such as pain, fatigue or self-efficacy, are also likely to influence intensity^42^; however, the relative contribution and interaction of these factors in determining intensity remain poorly defined. The nuance between quality and quantity of movement is unlikely to be recognised within spine care, where postoperative advice typically focuses on total movement rather than the effort at which movement is carried out. Understanding the distinction between PA volume and intensity is important, as targeted interventions may need to prioritise different drivers to enhance both the quality and quantity of postoperative activity.

In other orthopaedic populations, targeted physical activity interventions have successfully improved MVPA levels. Christiansen et al. demonstrated that a physical therapist-led programme, incorporating step-goal setting and monitoring, increased MVPA by up to 70 min per week at six months post knee arthroplasty, compared with standard care^43^. Similarly, Losina et al. showed in a RCT that combining financial incentives with telephone health coaching following knee arthroplasty yielded an increase of 25 min of weekly MVPA compared to controls^44^. To our knowledge, no studies have evaluated the effect of similar targeted interventions on MVPA in decompression cohorts, representing an important gap in spinal research.

### The function-activity gap

A recurring theme across the included studies was the lack of correlation between objectively measured physical activity and PROMs. It is important to distinguish between physical function, physical capacity, and physical activity: physical function describes self-perceived ability captured through PROMs, physical capacity represents the ability to perform activity under maximal effort, and physical activity reflects free-living behaviour. Following lumbar decompression, studies reported substantial improvements across key patient-reported outcomes, such as pain (VAS), disability (ODI) and health-related quality of life (SF-12)^22,28,29^. Sunderland et al.’s case series of 2699 patients similarly showed significant improvement in a range of patient-reported domains^45^. There is therefore strong evidence that decompression improves symptoms and self-perceived physical function.

There is also evidence to support that decompression improves objective physical capacity. In a cohort study of 38 patients undergoing decompression, Smuck et al. noted significant improvements in the Self-Pace Walking Test, a validated measure of PA capacity in the lumbar spinal stenosis population^29,46^. Similarly, Försth et al. found in a RCT that decompression yielded an increase of 80 m in the Six-Minute Walk Test - a result above the minimal clinically important difference^47,48^. However, in the present study, we did not find a marked increase in free-living physical activity. There is hence a clear dissociation between what decompression patients *feel* they can do, what they *can* do under maximum effort, and how they *actually* move in free-living conditions - a concept termed the function-activity gap^29^.

The emerging picture is concerning: despite gaining more capacity for physical activity, many decompression patients remain inactive in free-living. Given that pain and disability are significantly reduced following decompression, as demonstrated by improvements in Visual Analog Scale (VAS) and Oswestry Disability Index (ODI) scores, physical limitations alone are unlikely to explain the low activity levels observed. Instead, behavioural factors, such as inertia following prolonged inactivity and low self-efficacy, likely play a role. Bienstock et al. proposed that in this patient population, who are elderly and experience significant pain, decompression is viewed as a means of restoring comfort and autonomy, rather than a platform for increasing physical activity^18^. Therefore, while clinicians may regard higher activity as an important indicator of recovery, patients themselves may prioritise symptom relief, independence, or the ability to perform activities of daily living, as more important markers of recovery. Similarly, Inoue et al. found that baseline physical activity levels strongly predicted postoperative outcomes^25^. Taken together, these findings provide strong evidence that habitual behaviour and patient preferences are key determinants of recovery and postoperative PA in lumbar decompression patients.

Encouragingly, recent work suggests that behavioural change in this population is achievable. The Spinal Stenosis Pedometer And Nutrition Lifestyle Intervention (SSPANLI) trial, which provided patients with personalised counselling and activity targets, showed increases in mean daily step count and maximum continuous activity^49^. Although preliminary, these findings show that decompression patients are receptive to interventions aimed at increasing physical activity. Large, high-quality randomised trials are needed to develop and validate targeted interventions in post-surgical populations.

### Technical considerations

There was substantial methodological heterogeneity between studies. Research-grade accelerometers, such as ActiGraph (ActiGraph LLC Pensacola, FL) and StepWatch (Modus Health, Inc., Washington, DC), provide triaxial data that capture posture, movement intensity and sedentary behaviour with high resolution. In contrast, consumer-grade devices, such as Fitbit and smartphones, have shown poor agreement with accelerometer-derived measures and should be used with caution^50^. Differences in device capability likely contributed to the heterogeneity observed in our analysis.

Beyond device type, the choice of metric and where it is derived from influences measurement accuracy. Step count, the most commonly reported outcome, captures only a single dimension of movement and is poorly validated in individuals with gait abnormalities or variable walking speeds^50^- issues especially relevant in postoperative cohorts. Measurement accuracy is further affected by device placement, as wrist-, waist-, and ankle-worn monitors differ in their sensitivity to specific movement patterns^51^. In addition, monitoring duration impacts the reliability of derived estimates, with at least four valid wear days generally required to obtain representative measures of habitual activity^51^. Across the included studies, these methodological elements were applied inconsistently, with varying wear-time thresholds, placement sites, and outcomes. Given such variability, it is important to carefully plan monitoring protocols which yield valid, comparable estimates of physical activity.

### Limitations

This review has several limitations. First, the number of included studies was small, and most had modest sample sizes. As a result, the meta-analysis was underpowered to detect small effects and could not support subgroup or sensitivity analyses. Second, heterogeneity between studies was substantial, arising from variations in device type, wear location, wear-time criteria, and physical activity metrics. These methodological inconsistencies likely influenced the pooled estimates and may limit cross-study comparability. Third, several studies included mixed surgical cohorts, with decompression analysed alongside fusion. This limits the ability to attribute the observed results solely to decompression. Finally, the short follow-up duration of included studies precluded the assessment of long-term recovery trajectories.

### Recommendations for future work

Progress in the spinal-physical activity field will depend on the establishment of consensus-based standard operating procedures (SOPs) that define optimal device type, placement, wear-time thresholds, and data-processing approaches. Standardisation of monitoring protocols is essential to ensure accurate and reproducible measurements of physical activity across studies. A major evidence gap is the absence of long-term data on objectively measured PA after lumbar decompression. Future work should therefore prioritise large, longitudinal cohorts with extended follow up using research-grade accelerometers, focussing on decompression-only cohorts. These studies should also stratify patients by preoperative function and symptom duration, to identify thresholds beyond which functional recovery becomes unlikely. Once the determinants of PA are better characterised, high-quality randomised controlled trials are needed to define the timing, targets, and effectiveness of interventions designed to improve free-living physical activity.

## Conclusion

This systematic review and meta-analysis provides the first comprehensive synthesis of objectively measured physical activity after lumbar decompression surgery. Physical activity trended towards improvement at three and six months postoperatively, though this was not significant. As such, despite reductions in pain and disability, objectively measured behaviour fails to mirror subjective recovery, highlighting a persistent function-activity gap. While decompression restores physical capacity, surgery alone is unlikely to produce sustained improvements in free-living physical activity without additional rehabilitative interventions. Incorporating objective PA metrics into postoperative assessments represents an important evolution in how recovery is defined in spine surgery. Future research should prioritise longitudinal study designs incorporating both pre- and postoperative monitoring, standardised accelerometer protocols, and consistent reporting of activity intensity. Ultimately, integrating objective physical activity metrics alongside PROMs will provide a more complete assessment of surgical success, and refine rehabilitation strategies that maximise the functional capacity restored by decompression.

## Supplementary Information

Below is the link to the electronic supplementary material.

Supplementary Material 1

## Data Availability

The datasets generated during and/or analysed during the current study are available from the corresponding author on reasonable request.
