# Supplementary material for: Objectively measured physical activity following lumbar decompression surgery: systematic review and meta-analysis
Source: Sci Rep. 2026 Apr 1;16:15291. doi: 10.1038/s41598-026-44749-1 (PMC13180964; doi:10.1038/s41598-026-44749-1)

**Objectively Measured Physical Activity Following Lumbar Decompression:**

**Systematic Review and Meta-Analysis (SUPPLEMENTARY MATERIAL)**

Sree Kanakala^1*^, Alisha Mahmud^1^, Iihan Ali^1^, Hassan Tahir^1^, Riese Patel^2^, Milos Brkljac^1^, Tim Lindsay^1^

**INSTITUTION:**

1. Faculty of Medicine, Imperial College London, London, United Kingdom
2. Faculty of Medicine, University of Birmingham, Birmingham, United Kingdom

*** Corresponding author:** Sree Kanakala, Faculty of Medicine, Imperial College London, London, United Kingdom. Email: sree.kanakala21@imperial.ac.uk

**Contents**

*Supplementary Table 1:* Search Strategy

*Supplementary Table 2:* OCEBM Level of Evidence

*Supplementary Figure 1:* PRISMA Diagram

*Supplementary Figure 2:* Bubble Plot

*Supplementary Figure 3:* ROBINS-I Traffic Light Plot

*Supplementary Figure 4:* ROBINS-I Summary Plot

| ("Laminectomy"[MeSH Terms] OR "Diskectomy"[MeSH Terms] OR "Decompression, Surgical"[MeSH Terms] OR "Spinal Fusion"[MeSH Terms] OR "Arthrodesis"[MeSH Terms] OR "Arthroplasty, Replacement, Intervertebral Disc"[MeSH Terms] OR ((spine OR spinal OR cervical OR thoracic OR lumbar OR lumbosacral) AND (decompress* OR laminectom* OR laminotom* OR foraminotom* OR discectom* OR diskectom* OR microdiscectom* OR fusion OR arthrodes* OR replac* OR arthroplast* OR prosthes*)) OR TLIF OR PLIF OR ALIF OR LLIF OR OLIF OR XLIF OR ACDF OR PCF OR TDR OR CDR)  AND  ("Accelerometry"[MeSH Terms] OR "Actigraphy"[MeSH Terms] OR "Monitoring, Ambulatory"[MeSH Terms] OR acceleromet* OR actigraph* OR actimetr* OR pedomet* OR activpal OR "activ pal" OR stepwatch* OR axivity OR geneactiv* OR actigraph OR "inertial measurement unit" OR IMU OR gyroscop* OR "activity monitor*" OR "activity tracker*" OR wearable*)  AND  ("Motor Activity"[MeSH Terms] OR "Exercise"[MeSH Terms] OR "Physical Exertion"[MeSH Terms] OR "Sedentary Behavior"[MeSH Terms] OR "physical activ*" OR exercise* OR "motor activ*" OR step* OR "step count" OR "activity count*" OR MVPA OR "moderate to vigorous physical activity" OR LPA OR "light physical activity" OR CPM OR "counts per minute" OR "vector magnitude" OR ENMO OR MET OR METs OR "metabolic equivalent*" OR "energy expenditure" OR sedentary OR "sedentary behavior" OR "sedentary behaviour" OR sitting OR standing OR "postural allocation" OR "activity volume" OR "time spent") |
| --- |

**Supplementary Table 1. Table showing PUBMED search strategy**. The search consisted of three distinct concept blocks: (1) decompression-related spinal procedures, (2) physical activity monitors, and (3) physical activity measures. The same strategy was adapted for Scopus, EMBASE, MEDLINE, and CINAHL with syntax adjusted appropriately for each database. A deliberately broad strategy was employed to maximise sensitivity, encompassing decompression, fusion, and disc replacement terms. During screening, inclusion was subsequently restricted to studies reporting lumbar decompression cohorts, consistent with the review’s focus.

# **Supplementary Table 2: Level of evidence based on the Oxford Centre of Evidence-Based Medicine (OCEBM) Levels of Evidence.**

| **Study** | **OCEBM Level** |
| --- | --- |
| Aubry 2021 | 2b |
| Bienstock 2022 | 2b |
| Chauhan 2024 | 2b |
| Coronado 2021 | 2b |
| Inoue 2020 | 2b |
| Mobbs 2016 | 2b |
| Scheer 2017 | 2b |
| Schulte 2010 | 2b |
| Smuck 2018 | 2b |
| Stienen 2020 | 2b |

**Supplementary Figure 1. PRISMA diagram showing study workflow.** Number of records identified from each database can be found in ‘Studies from databases/registers (n = 1566)’.

**
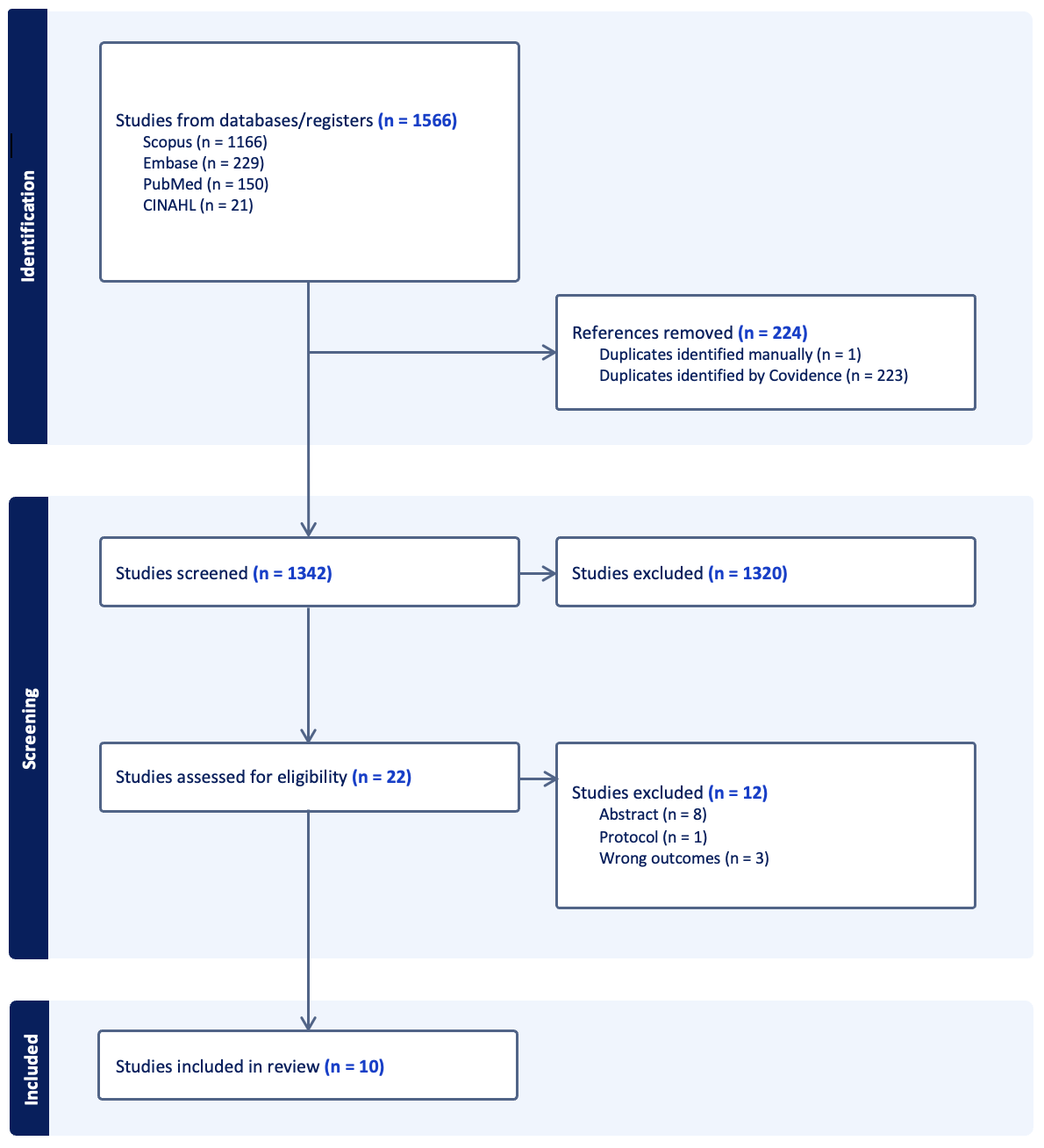
**

**Supplementary Figure 2. Bubble plot showing year of publication and sample size for included studies.** The plot shows consistent publications over the last decade.

**
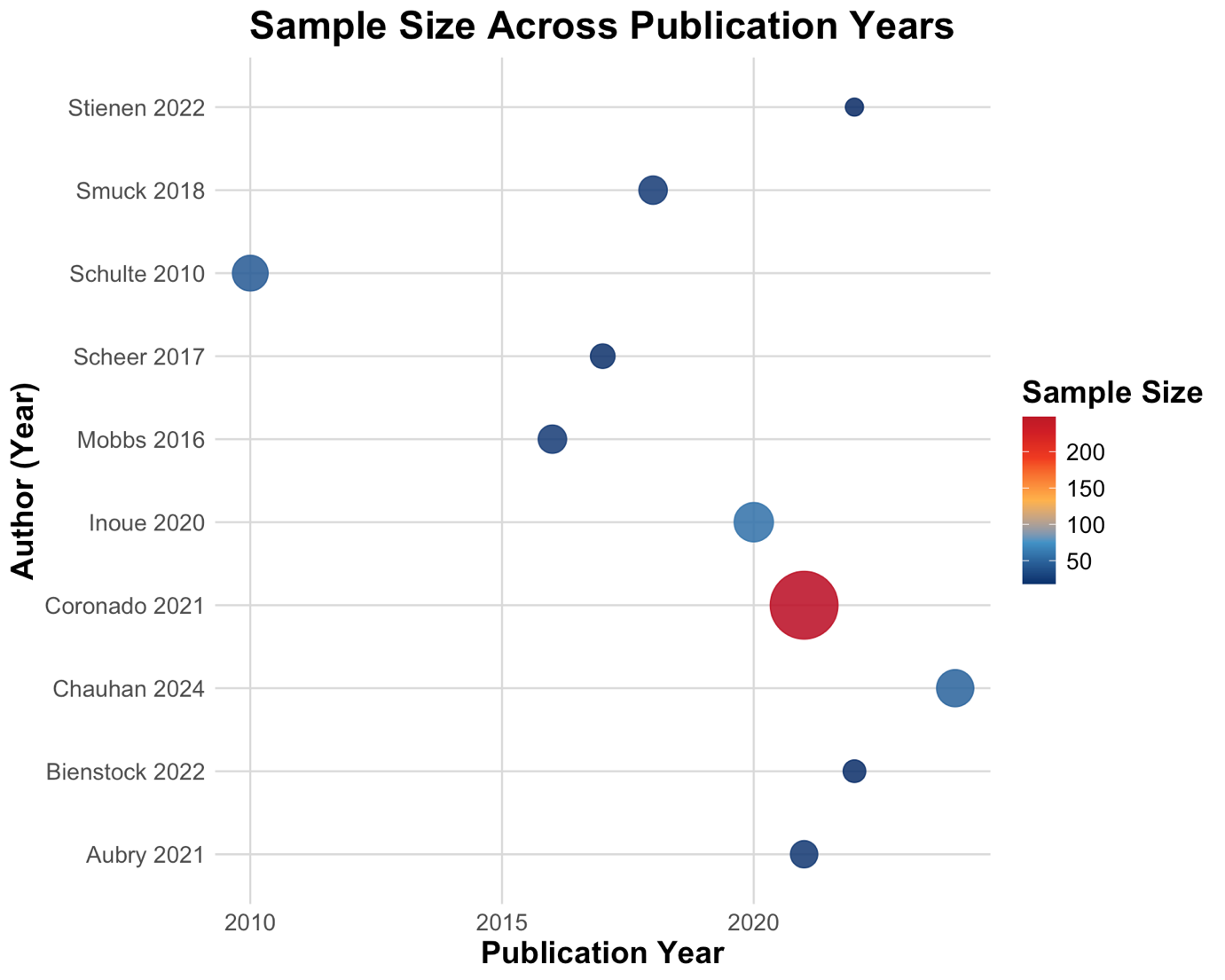
**

**Supplementary Figure 3. ROBINS-I traffic light plot for all included studies.**


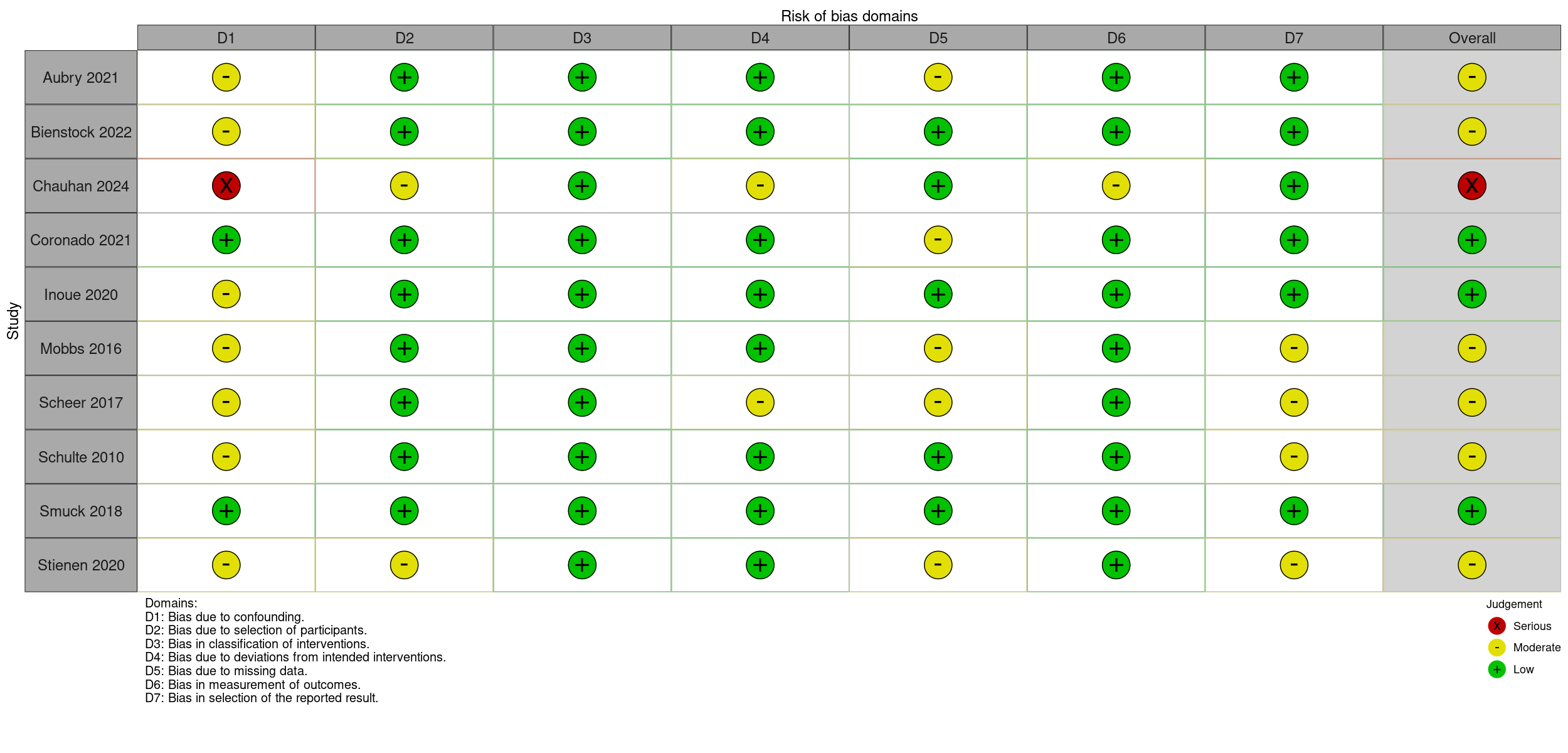


**Supplementary Figure 4. ROBINS-I summary plot for all included studies.**


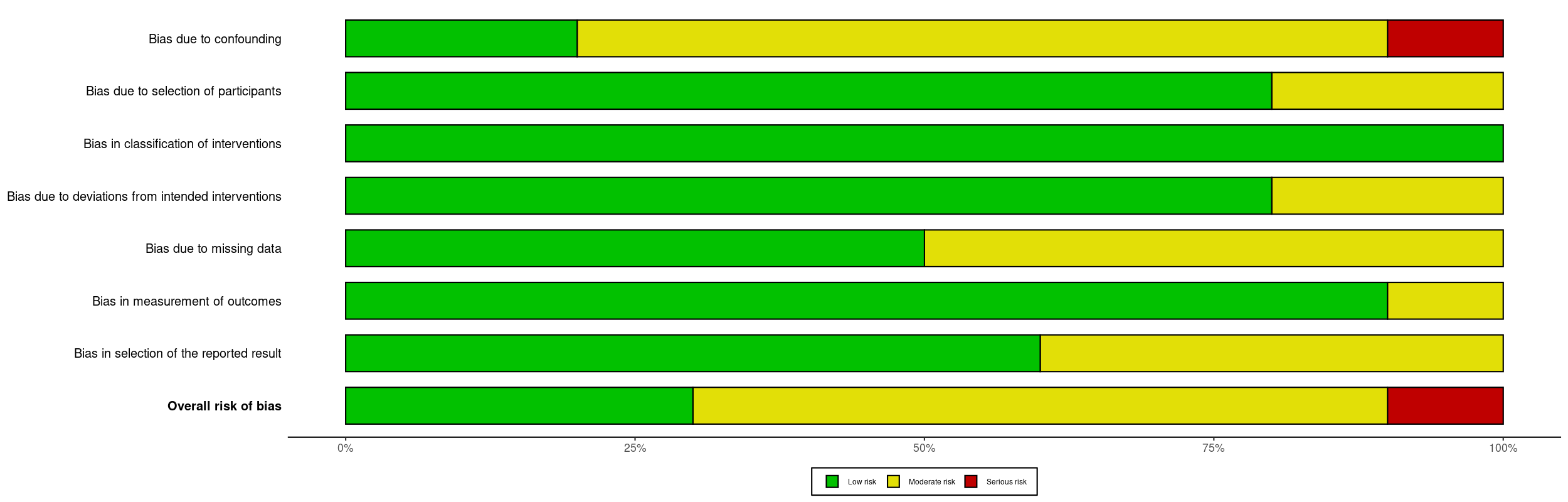

Supplement: Supplementary file 1 — Supplementary Material 1 [file 41598_2026_44749_MOESM1_ESM.docx]
